# A Rational Exposition of the Physical Signs of the Diseases of the Lungs and Pleura, &c.

**Published:** 1834-01-01

**Authors:** 


*A Rational Exposition of the Physical Signs of the Diseases of the Lungs and Pleura, illustrating their Pathology, and facilitating their Diagnosis.* By CHARLES J. B. WILLIAMS, M.D. *Second Edition.*—London, 1833. 8vo. pp. 201; and 2 plates.

THE theory of the stethoscope is perhaps the most splendid medical discovery of this century; and, did the practice but keep pace with it, the physician would no longer, in thoracic diseases at least, be compelled to repeat the lamentation of the Coan sage, and confess that "diagnosis is difficult." Unfortunately, however, there are few instances in which practice has followed theory with so limping a step as in the use of this ingenious instrument; so that, among the crowds who use the stethoscope, it would be difficult to point out a single tolerable stethoscopist,—that is, one who is able to determine with something like certainty the nature of the disease, when the ordinary symptoms are doubtful. Yet we would not have the medical philosopher despair; for he will perhaps find, before the lapse of many years, that a number of great hospital physicians will gradually be educated to the stethoscope, and will publish the results of their practice, exhibiting a host of cases in which the ordinary symptoms left them in doubt, but the stethoscopic indications enabled them to cure their patients. Moreover, we can assure the student that the habit of attention taught by the use of the stethoscope is in itself a great gain; so that, if he does not find the treasure he is seeking, at any rate, like the young husbandman in the old story, he will have fertilized the soil in which he is labouring.

Dr. Williams's work is a clear exposition of the theory of the art, and will be useful to beginners.

Why are there two pages 21, and two pages 22, in this little book?

